# The Effect of Respiratory Viral Infections on Breakthrough Hemolysis in Patients with Paroxysmal Nocturnal Hemoglobinuria

**DOI:** 10.3390/ijms24119358

**Published:** 2023-05-27

**Authors:** Ioanna Lazana, Sean Apap Mangion, Selma Babiker, Joanna Large, Roochi Trikha, Mark Zuckerman, Shreyans Gandhi, Austin G. Kulasekararaj

**Affiliations:** 1 Department of Hematological Medicine, King’s College Hospital-NHS Foundation Trust, London SE5 9RS, UK; 2 Cell and Gene Therapy Laboratory, Biomedical Research Foundation of the Academy of Athens, 115 27 Athens, Greece; 3 Department of Virology, King’s College NHS Foundation Trust, London SE5 9RS, UK; 4 Faculty of Life Sciences and Medicine, King’s College London, London WC2R 2LS, UK

**Keywords:** paroxysmal nocturnal hemoglobinuria, breakthrough hemolysis, respiratory virus infections

## Abstract

Paroxysmal nocturnal hemoglobinuria (PNH) is characterized by hemolysis and thrombosis and is associated with significant morbidity and mortality. Although complement inhibitors have significantly changed the outcomes in PNH patients, breakthrough hemolysis (BTH) may still occur as a response to stress factors such as pregnancy, surgery, and infections. Despite the well-described association between bacterial infections and hemolysis in PNH patients, little is known about the effect of respiratory viruses on triggering hemolytic episodes. This is the first study, to our knowledge, addressing this question. We retrospectively analyzed 34 patients with PNH disease between 2016 and 2018, who were on eculizumab treatment and who presented with respiratory symptoms and were subsequently tested for 10 respiratory viruses (influenza A, influenza B, parainfluenza, respiratory syncytial virus, adenovirus, rhinovirus, and human metapneumovirus). NTS+ patients had higher inflammatory markers, with the majority requiring antibiotics. Acute hemolysis, along with a significant drop in hemoglobin, was noted in the NTS+ group, with three of them requiring a top-up transfusion and two requiring an extra dose of eculizumab. Furthermore, the time from the last eculizumab dose was longer in the NTS+ patients who had BTH, than those who did not. Our data indicate that respiratory virus infections pose a significant risk for BTH in PNH patients on complement inhibitor treatment, underlining the need for regular screening and close monitoring of patients with respiratory symptoms. Furthermore, it implies a higher risk for patients who are not established on complement inhibitors, suggesting the necessity for greater vigilance in these patients.

## 1. Introduction

Paroxysmal nocturnal hemoglobinuria (PNH) is a rare hematological disorder associated with a clonal expansion of hematopoietic stem cells [1]. It is caused by a loss-of-function mutation affecting the X-linked *PIG-A* gene, which results in deficiencies of glycosylphosphatidylinositol (GPI)-anchored proteins [2], such as the surface membrane complement regulatory proteins CD55 [3] and CD59 [4], which are responsible for regulation of C3 and C5 convertase formation and stability and blocking the formation of membrane attack complexes (MAC), respectively, leaving cells vulnerable to complement attack [5]. While a wide variety of clonal cells will exhibit GPI-anchored protein deficiency, including platelets, polymorphonuclear leukocytes, monocytes, T-cells, and natural killer lymphocytes, erythrocytes are the most vulnerable to lysis as a result of their enucleated state [6,7].

Clinically, PNH is characterized by hemolytic anemia and thrombosis and is associated with significant morbidity and mortality [8]. Thrombosis constitutes the leading cause of death, occurring in up to 50% of PNH patients. Early diagnosis and treatment of an acute hemolytic process is critical, as hemolysis itself is a risk factor for thrombosis and organ damage [9]. Furthermore, there is a significant impairment in the quality of life in patients with recurrent or acute hemolytic episodes [10]. It is of particular importance that the propensity for complement-induced lysis is further exaggerated by various situations, both physiological, such as the intravascular acidic state occurring during sleep [7] and pregnancy, and pathological, such as trauma, surgical procedures, vaccinations, and infections [9,10]. Eculizumab is a long-acting humanized monoclonal antibody targeted against complement C5, inhibiting its cleavage into C5a and C5b. This, in turn, inhibits the activity of the terminal complement system including the formation of MAC. It stops hemolysis, but by inhibiting terminal complement activation, it increases susceptibility to encapsulated bacterial infections, especially *Neisseria meningitidis*. Common adverse events include infections such as pneumonia and respiratory infections [11].

With regard to infections, there are only two case reports in the literature, suggesting that viral infections may have a role in inducing acute hemolysis [12]. Furthermore, it has recently been reported that SARS-CoV-2 infection or COVID-19 vaccination may provoke breakthrough hemolysis (BTH) [13,14]. However, there are no studies investigating the possible association between viral infections and hemolysis in PNH disease. This is the first study to our knowledge describing a correlation between respiratory virus infections and induction or exacerbation of hemolysis in PNH patients on complement inhibition. Our study does not include any SARS-CoV-2 infection/vaccination-induced complement activation and hemolysis, as we are prospectively studying the impact of this phenomenon.

## 2. Results

### 2.1. Patient Characteristics

A total of 34 patients with PNH disease presented with fever and/or coryzal symptoms and were assessed for respiratory virus infection by NTS collection. Clinical characteristics, such as the type of PNH disease, indication and duration of eculizumab treatment, previous thrombotic or hemolytic events, transfusion status, use of antibiotic prophylaxis, and immunosuppressive treatment, were collected and are shown in Table 1.

**Figure 1:**
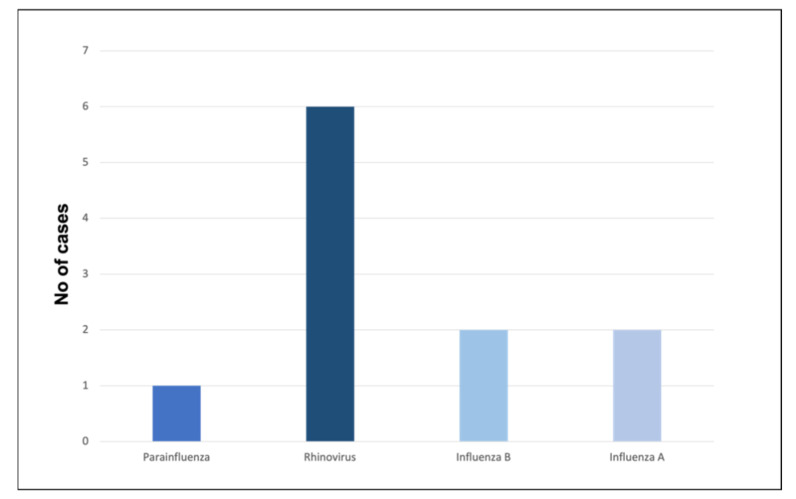
The figure illustrates the type and frequency of viruses detected in the combined nose and throat swabs.

A positive NTS result was detected in 11 patients (32%) and that included 1 parainfluenza, 6 rhinovirus, 2 influenza B, and 2 influenza A (H3N2) viruses (Figure 1).

The median age was similar between the NTS+ and NTS− groups. There was a female predominance in the NTS− group (17/23) and a male predominance in the NTS+ group (7/11) (*p* = 0.54). Most of the patients, in either group, were on prophylactic antibiotics, with only a minority of patients (1/11 and 3/23 for NTS+ and NTS−, respectively) not receiving penicillin V prophylaxis. None of the NTS+ patients were on cyclosporin at the time of the testing.

All 34 patients had been on eculizumab when tested. Of the 11 NTS+ patients, 7 had been on eculizumab treatment for hemolytic PNH and 3 for thrombosis related to PNH, whereas 22 and 0 NTS− patients had been on eculizumab for hemolysis and thrombosis, respectively.

The duration of eculizumab treatment appeared to be significantly longer in NTS+ patients compared to NTS− patients (mean 83.1 vs. 52.7 months, respectively, *p* = 0.0007). Five out of eleven (45%) NTS+ patients were transfusion dependent at the time of viral screening, in contrast to four out of twenty-three (17%) NTS− patients (*p* = 0.07).

### 2.2. Infection Parameters

The inflammatory response to viral infection was assessed by the WCC, neutrophil count and CRP, whereas the severity was evaluated by the need for antibiotic and/or antiviral treatment and the need for hospitalization. The results are shown in Table 2.

An increased CRP was noted in 6/11 NTS+ patients, in contrast to only 8/23 NTS− patients, and this rise was significantly higher in the NTS+ group (34.5% vs. 12.2%, in NTS+ and NTS− groups, respectively, *p* = 0.04). The majority of patients in the NTS+ group had a raised WCC, in contrast to 6/23 patients in the NTS− group. The percentage rise in the WCC was again significantly higher in the NTS+ group (33.8% vs. 11.1%, *p* = 0.01). Similarly, the percentage neutrophil increase was significantly higher in the NTS+ group (*p* = 0.04). No abnormality was noted in chest X-ray (CXR) in any of the NTS+ patients, whereas the blood cultures, performed in 7/11 patients, were all negative, in keeping with the diagnosis of a viral infection.

With regard to the requirement for antibiotics, over half of the NTS+ patients (6/11) received antibiotics, in contrast to only 5/23 (22%) patients in the NTS− group. Oseltamivir was used in 4/11 NTS+ patients that tested positive for influenza A or B. No difference in vaccination for seasonal influenza was seen between the two groups, although the overall uptake was low. None of the NTS− patients required treatment with GCSF for neutropenia, and only 1/11 NTS+ patients did.

### 2.3. Hemolytic Parameters

To assess the potent impact of viral infections in inducing/exacerbating hemolysis, i.e., BTH, various hemolysis parameters were analyzed and compared between NTS+ and NTS− patient groups, as illustrated in Table 3. More specifically, in the NTS+ group, 7/11 and 6/11 patients had a rise in LDH and bilirubin, respectively. In contrast, only 13/23 and 2/23 patients had increased LDH and bilirubin, respectively, in the NTS− group. More importantly, the percentage rise in bilirubin was significantly greater in the NTS+ group compared to the NTS− (mean 66.2 vs. 22.6, *p* = 0.02). DAT was performed in 4/11 and in 7/23 NTS+ and NTS− patients, respectively. Positivity was detected in 1/4 NTS+ patients (C3d 3+), whereas none on the NTS− patients had a positive DAT (C3d > 2+). Similarly, the majority of NTS+ patients (8/11, 73%) had a drop in Hb levels, compared to only 8/23 (35%) NTS− patients, with three requiring a top-up red cell transfusion to maintain a hemoglobin level of >80 g/liter (g/L). Again, the percentage drop in Hb was significantly higher in the NTS+ group, compared to the NTS− group (mean % drop 25.4 vs. 4.7, *p* = 0.002). In line with this, 2/11 (18%) NTS+ patients required an extra eculizumab dose to control hemolysis, whereas none of the NTS− patients required an added dose.

**Figure 2:**
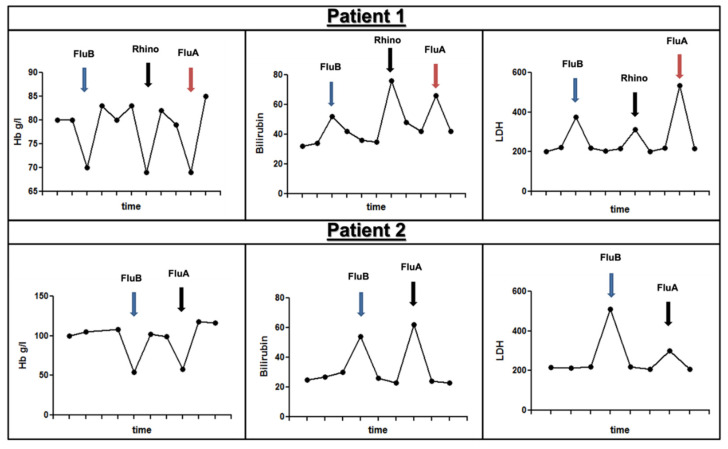
The figure illustrates a representative example of a patient who suffered three (Patient 1) and two (Patient 2) viral infections, respectively, in relation to hemolysis markers during the period analyzed. Each arrow indicates a different episode of viral respiratory infection. As depicted, each episode was associated with a significant drop in Hb levels, along with a rise in the bilirubin (umol/L) and LDH (U/L) levels. **Abbreviations:** Hb: hemoglobin, g/L: grams per liter, umol/L: micromole per liter, U/L: units per liter, LDH: lactate dehydrogenase.

Two of the eleven NTS+ patients had recurrent viral respiratory infections in the reviewed time period. Specifically, one experienced three (influenza B, rhinovirus, and influenza A) and the other experienced two (influenza A and B) infections (Figure 2). An associated significant BTH requiring red cell transfusion was seen in all patients. Furthermore, an extra dose of eculizumab had to be given to control the hemolysis in one patient.

## 3. Discussion

Hemolysis is a major clinical and diagnostic feature of PNH [5]. It has been implicated in end organ damage and in an impaired quality of life, resulting in fatigue, dysphagia, breathlessness, abdominal pain, and erectile dysfunction [15]. Furthermore, hemolysis alone can increase the risk of a—life threatening—thromboembolic event [16]. Finally, accumulating evidence suggests that ongoing hemolysis can be destructive, even in the absence of symptoms, underlying the need for early recognition and treatment [17]. Eculizumab has significantly improved the outcomes in PNH patients [18], leading to a reduction in hemolysis, transfusion requirement, and thrombotic events [9]. This is achieved by high affinity binding to C5 and inhibition of C5 cleavage, leading to terminal complement inactivation. However, it is widely recognized that any stress factor, such as pregnancy, surgical procedures, and infections, can lead to BTH, defined as hemolytic flares in PNH patients on complement inhibitors [19,20]. The only studies to date investigating the potential effect of respiratory viruses on inducing hemolysis are focused on SARS-CoV-2 infections. Our study is the first study to our knowledge that addresses this question and most importantly in the era of complement inhibitor treatment.

All patients included in our study had been on eculizumab treatment, the only approved anti-complement treatment in UK. At the time of testing for a viral respiratory tract infection, 11/34 patients had a documented respiratory virus infection. The demographic and clinical characteristics were similar between the NTS+ and NTS− patients. NTS+ patients had higher inflammatory markers, with the majority requiring treatment with antibiotics, whereas three patients required hospitalization for more intensive treatment. The antibiotic treatment was mostly empirical, given the raised inflammatory markers and the risk of secondary bacterial infections, especially in the immunocompromised setting, where blood cultures are commonly negative.

The majority of NTS+ patients had BTH with a significant rise in bilirubin and LDH levels and a significant drop in the Hb level. Interestingly, DAT was positive for C3d in ¼ of the tested NTS+ patients, indicating C3-mediated intracellular hemolysis. Three NTS+ patients required a top-up red blood cell transfusion to maintain their Hb level and two required an extra dose of eculizumab to control the hemolysis. These observations suggest that viral infections might be a significant trigger of breakthrough hemolysis, even in patients already established on eculizumab treatment, underlining the need not only for close monitoring, but also to educate PNH patients to promptly report and discuss any symptoms they may develop. NTS− patients presenting with respiratory symptoms may have either had a virus that was not included in the panel tested or have a misdiagnosis of a (viral) respiratory infection. Some of these patients (8/23) exhibited some degree of hemolysis, although not as prominent or as severe as the NTS+ patients, but again indicating the need for increasing awareness of the risks for BTH and prompt reporting of any possible viral symptoms.

It is also of interest the fact that NTS+ patients had been on eculizumab treatment for a significantly longer time compared to NTS− patients. Five out of eleven NTS+ patients continued to be transfusion dependent at that time. This may indicate a broader “deficit” in their immune response, leaving these patients more susceptible to (viral) infections and subsequently to hemolysis. This is in agreement with previous studies suggesting such a relationship [19]. Certainly, larger prospective studies are required to confirm the above, but should this be proven to be the case, it would allow earlier identification of patients who are at greater risk of infections and related complications, such as hemolysis, thrombosis, and end organ damage. Furthermore, in the NTS+ patients who exhibited some degree of hemolysis post viral infection, the time from the last eculizumab dose was longer compared both to those who did not and to the NTS− patients (mean 16.5 vs. 9.0 vs. 2.8 days, respectively), but this difference was not significant. This again is in keeping with previous data, suggesting an increased risk of BTH 1 to 2 days before the scheduled complement inhibitor dose [21]. Although most of these BTH occurrences were likely pharmacodynamic (PD) due to a complement amplifying condition, i.e., a viral infection, rather than pharmacokinetic (PK) due to subtherapeutic complement inhibition, we did not have complete data on drug levels, C5 levels, or CH50 to differentiate between PD versus PK BTH.

The limitations of our study were the inability to study the impact of SARS-CoV-2 on BTH, as the study was performed prior to the COVID-19 pandemic. Additionally, although our respiratory virus panel was comprehensive, it did not include SARS-CoV-2, parainfluenza 4, and other “seasonal” coronaviruses. We also did not have data on a comprehensive set of complement assays to differentiate PD and PK BTH. The impact of viral infections on patients treated with novel complement inhibitors, especially proximal component inhibitors, would be critically important in view of severe BTH due to a large population of PNH red cells [22].

Overall, our data indicate that respiratory virus infections may pose a significant risk for BTH in PNH patients, underlying the need for influenza and SARS-CoV-2 immunization, regular screening, and close monitoring of symptomatic and high-risk PNH patients, even those who are stable on complement inhibitor therapy. A significant degree of breakthrough hemolysis was noted in most of the patients despite all the cohort receiving eculizumab protective therapy at the time of viral infection. This has led us to wonder whether viral infections can be more detrimental in patients who are not on therapy with complement inhibitors, leading to an increased risk of subsequent life-threatening complications. Further, larger prospective studies are required to confirm the above and also systematically study the effect of SARS-CoV-2 and other respiratory viruses on BTH in patients with PNH.

## 4. Materials and Methods

We retrospectively analyzed 34 patients with PNH, treated with eculizumab at King’s College Hospital between September 2016 and March 2018. Depending on the symptoms, some patients had combined nose and throat swabs (NTSs) collected. Swabs were tested by multiplex polymerase chain reaction (PCR) for the following 10 respiratory viruses: influenza A, influenza B, parainfluenza (type 1–3), respiratory syncytial virus (RSV, subgroup A and B), adenovirus, rhinovirus, and human metapneumovirus.

Data were collected as part of a standard of care evaluation of patients with PNH disease presenting with respiratory symptoms. Various hematological and biochemical parameters were assessed. Specifically, hemoglobin (Hb) levels, reticulocyte counts (retics), lactate dehydrogenase (LDH), bilirubin, and direct antiglobulin tests (DAT) were used to assess for hemolysis. As infection markers, the total white cell count (WCC), the neutrophil count, and the C-reaction protein (CRP) were evaluated. Radiological evaluation was requested as appropriate.

Depending on the clinical assessment and results of the parameters above, the need for hospitalization, treatment with antibiotics and/or antivirals, extra complement inhibitor (eculizumab) therapy, or a top-up red cell transfusion was evaluated.

### Statistical Analysis

For analyses, a Student’s t test and Fischer’s exact test were used. The results are described either as means and standard error of the mean (SEM) or medians. *p* < 0.05 was considered significant. Statistical analyses were performed with IBM SPSS version 26.

## Figures and Tables

**Table 1:** Baseline characteristics of patients. They are divided into two categories based on whether they screened positive (NTS+) or negative (NTS−) in the respiratory virus screening.

Characteristics	NTS+	NTS−	*p*-Value
**Number of patients**	11	23	
**Age, years (median)**	49.5	51	
**Sex**			
Male	7	6	
Female	4	17	
**Classification of PNH**			
Classical	7	19	
Associated with other BM disorder	4	4	
**Indication for Eculizumab Rx**			
Hemolysis	7	22	
Thrombosis	3	0	
Other (pregnancy)	1	1	
**Eculizumab duration of Rx months, mean (SEM)**	83.1 (16.78)	52.7 (6.28)	0.0007
**Antibiotic prophylaxis**			
Yes	10	20	
No	1	3	
**On Cyclosporin**			
No	11	21	
Yes	0	2	
**Transfusion Dependent**			0.07
Yes	5	4	
No	6	19	

**Abbreviations:** NTS: nasal and throat swab, PNH: paroxysmal nocturnal hemoglobinuria, Rx: treatment, SEM: standard mean of error.

**Table 2:** Infection parameters in NTS+ and NTS− patients.

Characteristics	NTS+	NTS−	*p*-Value
**CRP rise**			
Yes	6	8	
No	5	15	
**Percentage increase in CRP (mean, SEM)**	34.5 (9.7)	12.2 (3.4)	0.04
**WCC rise**			
Yes	8	6	
No	3 *	17	
**Percentage increase in WCC (mean, SEM)**	33.8 (7.1)	11.1 (4.7)	0.01
**Neutrophil rise**	8	6	
**Percentage increase in neutrophil count (mean, SEM)**	42.8 (14.1)	12.7 (5.5)	0.04
**Neutrophil drop**	3	8	
**Percentage drop in neutrophil count (mean, SEM)**	39.07 (24.37)	20.05 (3.06)	0.22
**Hospitalization**			
Yes	3	2	
No	8	21	
**Need for Antibiotics**			
Yes	6	5	
No	5	18	
**Need for Oseltamivir**			
Yes	4	0	
No	7	23	
**Need for GCSF**			
Yes	1	0	
No	10	23	

* In 1/3 patients, there was a drop in the WCC and neutrophil count. **Abbreviations:** NTS: nasal and throat swab, CRP: C-reaction protein, SEM: standard mean of error, WCC: white cell count, GSCF: granulocyte-colony stimulating factor.

**Table 3:** Hemolysis markers in NTS+ and NTS− patients.

Characteristic	NTS+	NTS−	*p*-Value
**LDH increase**			
Yes	7	13	
No	4	10	
**Percentage increase in LDH (mean, SEM)**	87.9 (28.4)	51.6 (14.5)	0.2
**Bilirubin**			
Yes	6	2	
No	5	21	
**Percentage increase in bilirubin (mean, SEM)**	66.2 (13.7)	22.6 (3.0)	0.02
**Hemoglobin drop**			
Yes	8	8	
No	3	15	
**Percentage drop in hemoglobin (mean, SEM)**	25.4 (4.5)	4.7 (1.6)	0.002
**Need for RBC transfusion**			
Yes	3	0	
No	8	23	
**Extra eculizumab dose**			
Yes	2	0	
No	9	23	
**Time from last eculizumab dose (days) at NPA testing (mean, SEM)**	11.2 (5.2)	2.8 (1.2)	0.53

**Abbreviations:** NTS: nasal and throat swab, LDH: lactate dehydrogenase, SEM: standard mean of error, RBC: red blood cells.

## Data Availability

Data available on request due to restrictions.
